# Experiments and Observations on Mesmerism

**Published:** 1829-08

**Authors:** Richard Chenevix


### MESMERISM.

*Experiments and Observations on Mesmerism.* By Richard Chenevix, Esq. F.R. and E.S., M.R.I.A., &c. (3d Article.)

The opinion of many who have devoted themselves to the study and practice of mesmerism is, that this art should be wholly confined to the benevolent end of curing diseases. This maxim is no doubt founded on charity, but it is satisfactory only as far as therapeutics are concerned; and more than one great province of mesmerism would lie uncultivated, were it strictly adhered to.

Mesmeric phenomena, in the present state of our knowledge, may be classed under three heads: pathological, physiological, and psycological. Humanity, perhaps, in its limited sense of individual good, might demand no more than an acquaintance with the former; but a wider spirit of advantage, that which makes men feel that the researches of philosophy are of higher import than the alleviation of many a malady, will desire investigations conducted upon other principles, and directed to every ramification into which this old, but oft rejected, truth can branch out. It is fortunate that, in pursuing the subject with a view to immediate utility, its other departments cannot be entirely neglected; and, in the hands of those who consider it merely pathologically, other phenomena start into notice, and claim attention when they are least expected.

Of all these phenomena, the most worthy of consideration are those denominated psycological. They are also the most rare, and, as far as my observation reaches, they are not strikingly manifest in more than three or four cases out of a hundred. They consist in a peculiar state of the mental faculties; and to it the name of lucidity has been applied. As, in the present article, no allusion will be made to any of these, it is useless to describe them here. Persons who desire more ample information may obtain it by consulting the foreign works, (for I am sorry to say there are none in English that treat of this subject,) and of which an enumeration was made in a preceding article.

Physiological phenomena are of much more frequent occurrence. They consist principally in a peculiar state of the nervous system, without reference either to the state of the mind or to the cure of diseases. They manifest themselves in mesmeric sleep, in particular sensations excited or allayed in the patient, by the action of the operator; in the suspension or the promotion of certain functions of animal life, whether voluntary or involuntary; certain modifications of the senses, contrary to what are admitted as the usual laws of nature, and produced entirely by the mesmeric influence. These are the most common results of mesmerism; and so frequent, indeed, that it may be questioned whether two animated bodies ever can come within certain limits without producing some of them, provided the nervous system of the one be but actively directed toward the other. These effects, it is true, may not be perceptible unless the nervous systems of both parties are suitably disposed; but they are not the less real on that account. As well might we say that the innumerable hosts of stars, discovered by later instruments, were created on the very day when Herschel first beheld them, as deny the existence of phenomena when so minute as to elude our senses, though we acknowledge their reality when they appear before us in more palpable forms.

To produce these phenomena, to make them evident to all who are attached to scientific truth, appears to me as legitimate a pursuit as any within the domain of physiology, provided it be held subordinate to the rules which should guide all similar researches, and the first of which is, that no experiment should be attempted if attended with unnecessary pain or danger. Since I began to occupy myself seriously upon the subject, not a single accident has occurred to me: neither is it probable that any will occur to operators who do not venture imprudently.

These observations were necessary, because much of what is to be stated in this article relates to the physiological class of mesmeric phenomena. Being desirous to prove by experiment that an assertion made in my first communication upon this subject is founded on fact, "*Mesmerism is true!*" I sought for an opportunity to put the science to the test, in some of the many hospitals with which this city abounds. By the kindness of Dr. Whymper, surgeon-major to the Coldstream regiment of Guards, and of Mr. G. Smith, surgeon to the same regiment, the first trial was made in their establishment; and an account of these experiments shall fill the following pages.

My short stay in London did not permit me to undertake any patient in the absolute hope of effecting a cure. Neither could I expect, in so short a time, to spread conviction very far. All my ambition was to excite curiosity; to break the ice of public incredulity; to turn the attention of a few of my eminent countrymen to a subject, of which so many distinguished foreigners have long admitted the truth. The most rapid phenomena, those which manifest themselves in the shortest time, were the most likely to conduce to this purpose; and with this intention the following experiments were undertaken.

The mode of proceeding was constantly as follows: Dr. Whymper and Mr. Smith examined their list of sick, and called into the room whomsoever they pleased. I had no communication whatever with the patient submitted to experiment but in their presence; and it very rarely happened that I uttered one single word to any of them previously to the operation, or while it lasted. The minds of all were entirely unprepared, at least until the process had become a subject of conversation among the inmates of the hospital; and then their opinions must have taken their bias from effects already produced, that is to say, from mesmerism.

April 12th.—Mr. Smith only was present this day, Dr. Whymper being occupied elsewhere. *Serjeant Oakley:* mesmerised him twenty minutes; no sensible effect: tried him no more.

*Case of Richard Ireland.*

April 12th. Mr. Smith alone present.—In two minutes' mesmerization, Ireland's eyes began to water, and his left nostril to run. His eyelids trembled very much. In six minutes I saw he was asleep. I then raised his arm almost as high as his head, and let it suddenly fall: he did not waken. When he had slept twenty-five minutes, I attempted to waken him by transverse passes made in that intention. He remained asleep, however, until I called him by name.

Second; April 14th. Dr. Whymper and Mr. Smith present.—In about five minutes he was asleep, and remained motionless for thirty minutes; at the expiration of which, I called him by name, and he awoke. Questioned by Dr. Whymper, he said he had slept soundly.

Third.—Ireland having been thus put to sleep by my mesmeric action, and in a manner which left little doubt upon the minds of Dr. W. and Mr. S., I was anxious to prove to these gentlemen a truth, which is one of the most important features of the doctrine, viz. that the faculty of mesmerising is not a peculiar gift, confined to a few "quos equus amavit Jupiter," but a power diffused as equally as any other power over the whole species. Serjeant Bradbury, who for nine years has superintended the pharmaceutic department of the hospital, was called in. I showed him the mode of operating, and gave him the necessary instructions, (which preliminaries may have occupied two minutes,) and he then proceeded to act. In six minutes Ireland was fast asleep. While he was in that state, I desired Serjeant Bradbury to raise his (the patient's) arm, nearly as high as his head, and let it suddenly fall. He did so: Ireland remained fast asleep. Bradbury, by my direction, attempted to waken him by transverse passes, but in vain. His name was then called, and he awoke. Questioned by Dr. Whymper, he said he was not aware that his arms had been raised or touched; that he had slept soundly, but had not been in the chair more than ten minutes.

Fourth; April 17th. Dr. W. and Mr. S. present.—Bradbury put him to sleep in five minutes very soundly. He remained motionless for twenty-five minutes, and was then awakened. Questioned by Dr. Whymper, he said he felt his health better, that he coughed less; did not know why he slept; was never in the habit of sleeping during the day, but supposed that the hands passing before his eyes, and the quiet of the room, set him to sleep.

Fifth; April 18th. Dr. W. and Mr. S. present. Mr. North was also present this day.—Bradbury put him to sleep in three minutes, and very soundly. He slept until Mr. North raised his arm, and let it fall suddenly. It is here necessary to state, that no person should be permitted to touch the patient while asleep, except the mesmeriser; the most dangerous consequences might ensue: but, as this man was not particularly sensitive, and as the gentlemen present were anxious to make some trials of their own, I consented, in order to satisfy them; warning them, at the same time, that such experiments should not be repeated.

Sixth; April 21st.—He was mesmerised by Bradbury, I not being present this day. A person, who witnessed this day's sitting, told Ireland to try to resist sleep. He did so, and succeeded; but his eyes and nose watered much, and the inclination to sleep was very great. He said that, had he shut his eyes one moment, he must have slept.

Seventh; April 22d. Dr. W. and Mr. S., with two private gentlemen, were present. Bradbury put him to sleep in six minutes, he being told not to resist. At the end of twenty minutes I woke him, by making transverse passes with that intention.

Eighth; April 23d. Dr. W. and Mr. S. present.—Bradbury mesmerised him for twenty minutes; but, as he said that he came into the room fully determined to resist, he did not sleep. His health was not improving, and no more experiments were attempted on this man.

The third patient tried here was *Garrand*, of the band of the regiment.

First; April 15th.—He did not sleep, though mesmerised thirty minutes, and said he was not aware of any effect upon him. I then tried the following experiment: Having previously announced to Dr. W. and Mr. S., out of the reach of the patient's hearing, that my intention was to communicate to Garrand's hand a sensation of heat or of cold, according to my will, without giving him any intimation of that will. I touched his hand with a silver pencil-case, with that intention. The results of the six first experiments were perfectly correct: that is to say, he felt the pencil-case cold when I willed that he should feel it cold, and hot when I willed that he should feel it hot, without committing a single mistake; but, when the experiment was often repeated, he began to err, and his sensations ceased to be according to my will. I must here state, that I have made this and similar experiments, with more or less success, at least upon eighty patients; and that I have always found that, when I repeated them too frequently at the same sitting, the tact of the patient, however accurate in the first trials, became as it were bewildered, and no longer distinguished the sensations according to my will. These anomalies have been observed by every practical mesmeriser, and are still more striking in the class of phenomena which I have above designated psycological. How to account for them, or for any of the effects of mesmerism, I know not; but such deviations from regularity are not uncommon in physiology. When we take successively into our mouths two known liquors, of different flavors, we immediately recognize them: when we repeat the trial too often, they are no longer distinguishable.

The following experiment was now performed: Garrand's eyes were most strictly blinded; he was desired to raise both his arms, and, being asked whether he felt any thing in either of them, he answered "No." A piece of paper, weighing perhaps from one to two grains, was placed upon his right sleeve, in such a manner that it was utterly impossible for him to feel it. He was then desired to raise both his arms, and was asked "Do you feel any thing?" "Yes." "What?" "A stiffness and weight in my right arm."

The same experiment was tried upon his feet, and with similar success, until too often repeated. Trial was then made whether he felt weight or stiffness in any of his limbs when the paper had been taken off without his knowing it: he felt none.

Second; April 16th.—He did not sleep, and evidently struggled much to avoid sleeping. His eyes, however, were so closed that, although he made many efforts to open them, he could not. He had a violent pain in his forehead, which some passes, made with an intention to relieve it, soon dissipated. The experiment of the pencil-case, tried by Dr. Whymper himself, succeeded as yesterday. That of the paper was not so satisfactory.

Had my intention been to follow up the treatment of this patient, and to attempt a further development of his mesmeric susceptibility, I should have kept him entirely under my own guidance; but, my object being to produce some prompt and striking result, the best method appeared to me to request Dr. Whymper to substitute his will and action in place of mine; for in this case he must be perfectly secure against collusion.

Third; April 17th.—Mesmerised him twenty minutes, but did not try any further experiments on him.

Fourth; April 18th. Dr. W. and Mr. S. present; Mr. North also present.—No sleep, but his eyelids were so much affected that he was two minutes at least struggling in vain to open them. After I had secretly announced to the gentlemen present that my intention was to make his limbs stiff and heavy by my action, I did so. It was acknowledged that this man could not have the slightest notion of what my intention was; consequently, that his mind could have no share in the effect. I then made a few transverse passes with an intention to set his limbs free, and they were free. The success of these experiments, upon frequent repetition, was not constant, and for reasons already stated; but the general result was acknowledged to be very extraordinary. I asked Mr. North, "Do you think these effects real?" "Yes." "Do you think they proceed from my action on this man?" "I can see no other cause."

Fifth; April 22d. Dr. W. and Mr. S., with two private gentlemen, present.—No sleep; was but slightly affected by the experiments above stated of the paper and the pencil-case. I fear they had been too frequently repeated the day before. I then said to the gentlemen present, (of course, without a possibility of being overheard by the patient,) "I will try to fix this man in his chair." I mesmerised him with that intention for about three minutes, and then said, in my usual tone of voice, "That will do for today; you may go." He rose from his chair like a man labouring under severe lumbago, and with considerable difficulty. Questioned by Dr. Whymper as to what prevented him from rising from his chair, he said, "My back and thighs are so stiff!" I then demesmerised him for about one minute, and he said the pains were gone; he added, "I felt as if a weight had been pulling me down."

The fourth patient was named *Simpson*. When he entered the room, Dr. Whymper asked him what he expected from the operation? he replied that he did not know, but that the other men said it numbed their blood. He was, to all appearance, asleep in ten minutes, with his head reclining backwards. After thirty minutes, he was asked how he felt? "Very odd; but I have not been asleep." "What do you mean by very odd." "The gentleman's hands seem to go through my flesh."

The fifth patient was *Mrs. Whitaker*, a soldier's wife, native of Bruxelles; very nervous, headachs, earachs, noises in her head. Dr. W. and Mr. S. present. She was so much alarmed at the idea of the operation, that she could hardly be calmed. Mesmerised her thirty minutes: no sleep. She said, however, of her own accord, that she had felt drowsy, and indeed that she had been nearly asleep three times, but was too much frightened to allow a fair result."

Second; April 18th. Dr. W. and Mr. S. present; Mr. North also present.—She was mesmerised about twenty minutes: appeared exhausted and sleepy during the operation, and said that she had been asleep. When she rose from the chair, she was reeling and tottering from side to side, and supporting herself upon every piece of furniture within her reach. She complained of great giddiness. A few transverse passes, made with an intent to demesmerise her, removed these sensations in less than half a minute. I then put the following questions to Mr. North: "Are not these phenomena extraordinary?" "They are." "Do you think they result from my action upon this woman?" "I do."* The other gentlemen, who had now become a little familiarised with mesmerism, were still more convinced of the reality of the effects which they saw.

When this woman was desired to sit again the next day, she objected, saying that it made her giddy, and confused and drowsy for the whole day, and that her child (she was pregnant) moved violently. She was evidently very nervous, and her imagination was much excited. She said, however, that the rheumatic pains of her head and face were much relieved.

The sixth patient was *Richard Gould*. April 22d. Dr. W., Mr. S., and two private gentlemen, present.—His complaint was rheumatism in the right thigh; he was

* It was not my intention to express my belief that the "transverse passes" removed the sensations of which the woman complained. I have no doubt she would have quickly and completely recovered from the sensations Mr. Chenevix describes, without any attempts to "demesmerise her." She had been, in fact, in a state very nearly resembling sound and natural sleep, and a few moments were required before she could regain her self-possession.—J. N.

selected promiscuously from a long list of patients. He slept in about three minutes, and remained motionless for half an hour. His arms were raised, as in the case of Ireland, and then let to fall, without any consciousness on his part. Transverse passes were made before his face, with an intention of wakening him, and in one minute he awoke. Questioned by Dr. Whymper, he answered that he did not know that his arm had been raised, or that any thing had been done to him; said that he felt his right thigh warmer than the other. He was not sensible to heat or cold produced by the contact of the pencil-case. A piece of paper placed upon his sleeve, as in experiments above related, gave him a sensation of weight and stiffness in the arm on which it rested; and he once complained of pain at the insertion of the deltoid muscle. He said, also, that his eyelids felt heavy and numb. A few transverse passes were then made, after which he declared that he felt quite recovered.

Second; April 23d. Dr. W. and Mr. S. present.—He was soundly asleep in one minute and a half. His arms were raised and let fall, without his knowing it. In twenty minutes, I woke him by transverse passes made before his face. I then put him to sleep again, and proposed to Dr. W. and Mr. S. to try upon him an experiment which is one of the most striking that can be performed upon patients who are affected by somnolency alone, without manifesting any other phenomenon. It is as follows: A patient being in complete sleep, the mesmeriser, without his knowledge, goes behind him, and there, at a given moment, makes transverse passes with an intention to awaken him. In such circumstances there can be no collusion between the operator and the operatee; neither can the imagination of the latter be anywise suspected of producing the result. It is an experiment which I have repeated before witnesses upon a great number of subjects, and very frequently, but which succeeds only upon persons endowed with great mesmeric susceptibility. In the present case the experiment was thus made: Having announced my intention to Dr. W. and Mr. S., without the possibility of Goold's being apprised of it, I took my station behind his chair. I then waited a couple of minutes, when I began to operate. At the very first pass his eyelids fluttered, and in about one minute he sat up erect in his chair. He soon became entirely awake, and in the same state as that in which he had been put by the previous operation.

Third; April 24th. Dr. W. and Mr. S. present.—Goold came into the room today evidently determined to resist, and twenty-two minutes elapsed before I could produce any effect upon him; neither should I have done so then, had I not told him that I was aware of his intention. His sleep, too, was not sound; and I regretted most particularly that I was thus prevented from fairly repeating the experiment of yesterday. My wish was to awaken this man again to-day by transverse passes made behind his back; but no sooner had I ceased to mesmerise him before his face, than he awoke. I mention the experiment of yesterday, then, more as one of which I myself am entirely convinced, than as one to the validity of which the medical gentlemen, who witnessed it but once, can give their full assent.

The seventh subject was *J. Fuller,* suffering from chronic dysentery. April 15th; Dr. W. and Mr. S. present. He was taking a grain and a half of opium daily.—No sleep, but felt his head unusually light and giddy; felt very comfortable. He was not sensible of any effect produced either by the pencil-case or the paper. He sat constantly with his eyes closed, and, when desired to open them, he was full two minutes before he could do so. When asked, "What prevented you from opening your eyes?" "I could not; they were fast closed; there was no strength in my eyelids; I never felt so before in my life."

This man was mesmerised again the next day, but only for a few minutes, and without any remarkable effect.

In a preceding article inserted in this Journal, it was stated that about one person in six was susceptible of mesmeric sleep; but, sleep not being the only effect, a larger proportion than this may manifest some of the phenomena. In fact, to eyes accustomed to observe the results of mesmerism, more than half of the subjects submitted to experiment show a certain susceptibility, in some shape or another. But, in the present state of the science, it would be unfair to obtrude upon novices the questionable phenomena which inexperience may hesitate to acknowledge, however evident to old practitioners. In every art tyros fail to discern the delicate touches on which their satisfaction is afterwards founded. For this reason I forbear to insist upon any results which did not afford entire conviction to the medical gentlemen who beheld them for the first time.

Seven patients, then, were mesmerised at the Coldstream Guards' hospital; three of whom, Ireland, Goold, and Mrs. Whitaker, slept. Every one of the seven, Oakley alone excepted, exhibited phenomena which no mesmeriser could for a moment mistake; which even struck Dr. W. and Mr. S.

as most extraordinary, and for a large portion of which it is impossible to account upon any known principle. A single example is sufficient to establish a general mode of reasoning upon all. Garrand, for instance, did not sleep, but he was susceptible of other sensations, as in the experiments of the pencil-case and of the paper, and when he was nearly rendered incapable of rising from his chair. Had this man been inclined to deceive us, the only effect of which he could have heard when first I mesmerised him, was sleep. This, then, was what he would have counterfeited, for he knew of no other; but he did not do so. The first time I touched his hand with the pencil-case, he had no more idea of what my intention was than if he had never seen me; yet the sensation which he described not only was one of temperature, and never of any other physical quality, as weight, hardness, &c., but it most accurately corresponded with the variations of heat and cold which, without his knowledge, I had announced as my will to two unbiassed and enlightened observers, both leaning rather toward the sceptical than toward the credulous side of philosophic hesitation. In the same manner, Garrand never felt the paper hot or cold, but always light or heavy; and light or heavy in exact conformity to my will, previously and distinctly announced to credible witnesses. Had this man, too, been the only one of these patients on whom similar effects were produced, something might be attributed to accident, to a casualty of his temperament; but Goold was hardly less susceptible than he was: Mrs. Whitaker manifested analogous symptoms; and, in all, six out of seven patients experienced sensible and undeniable effects. Neither does the diminution of sensibility or of accuracy in these experiments invalidate their reality: it confirms it; because such a diminution is in perfect harmony with all the laws of physiology. The galvanic excitability of the dead frog is exhausted by repetition, and too frequent stimulation extinguishes even life.

Since these experiments were terminated, others have been made at three hospitals in London, and at my own house, in presence of some very eminent men. An account of them shall be given successively in this Journal. Conviction, of course, has reached these persons in different degrees; and, lest the opinion of any one of them should be attributed to any other, I shall forbear mentioning them until the exact words of each are attached to his name. In what I have to communicate, I shall state nothing which has not been submitted to the gentlemen whose testimony I adduce, for their approbation; and very often I shall be able to quote their letters to me on the subject. My intention is to give, without reserve, and equally, an account of all the impressions, favorable and unfavorable, made by these experiments, and then to sum up the evidence on either side. In the present instance, the notes were taken, during each sitting, by Dr. Whymper himself, who communicated them to me; and from them I have drawn up the preceding narrative, adding only a few observations, which habit enabled me to make, and such explanations as appeared necessary to elucidate this unstudied subject.

---

### HOSPITAL REPORTS,

*(Principally condensed from various Periodical Publications.)*

---

### ROYAL WESTMINSTER OPHTHALMIC HOSPITAL.

*Report of Cases, attended by* Mr. J. Foote, Jun.; *under the Direction of* Mr. Guthrie.

